# Impact of Oxidative Stress-Driven Ferroptosis in Neurodegeneration

**DOI:** 10.3390/ijms27083353

**Published:** 2026-04-08

**Authors:** Asma Rafique, Aleena Junaid, Marica Bakovic

**Affiliations:** Human Health Sciences, University of Guelph, Guelph, ON N1G 2W1, Canada; arafique@uoguelph.ca (A.R.); ajunai01@uoguelph.ca (A.J.)

**Keywords:** ferroptosis, neurodegeneration, iron homeostasis, lipid peroxidation, GPX4, oxidative stress

## Abstract

Ferroptosis is an iron-dependent cell death driven by lipid peroxidation and failure of cellular antioxidant defenses. It is triggered by oxidative stress and can be aggravated by aging, inflammation, and dysregulation of iron homeostasis. In the central nervous system, iron dyshomeostasis, mitochondrial dysfunction, and membrane lipid remodeling can amplify oxidative injury and increase susceptibility to ferroptotic damage, particularly in vulnerable neurons. There is growing evidence that ferroptosis-related processes are linked to Alzheimer’s disease, Parkinson’s disease, Huntington’s disease, and Amyotrophic Lateral Sclerosis. This review addresses novel approaches to track ferroptosis in vivo, such as imaging and biomarker techniques, and important molecular mechanisms linking iron metabolism, reactive oxygen species, and PUFA-driven lipid peroxidation to neuronal damage. We also explore upstream transcriptional control via NRF2, iron chelation and iron-handling modulation, inhibition of lipid peroxidation, and reinforcement of the System Xc-GSH-GPX4 and CoQ10-linked defense pathways. Subsequently, we highlight translational issues that need attention to further progress ferroptosis-targeted therapies for neurodegenerative disease.

## 1. Introduction

Ferroptosis is a regulated form of cell death that is dependent on iron. It is mainly caused by extensive accumulation of lipid peroxides, particularly phospholipid hydroperoxides, which develop from oxidative stress and are distinct from other forms of cell death [1,2]. Ferroptosis plays a major role in the pathogenesis of various diseases such as cancer, ischemic organ injury, cardiac, and neurodegenerative diseases [3,4]. Its unique biochemical features and regulatory pathways position ferroptosis as both a driver of disease progression and a potential therapeutic target.

The central nervous system (CNS) is particularly susceptible to ferroptotic damage due to its high metabolic activity, elevated oxygen consumption, and abundance of polyunsaturated fatty acids, which are highly prone to oxidative modification. In addition, the brain requires tightly regulated iron homeostasis for essential processes such as neurotransmitter synthesis and mitochondrial function. Disruption of this balance can contribute to neuronal vulnerability and has been increasingly associated with neurodegenerative conditions such as Parkinson’s disease (PD), Alzheimer’s disease (AD), Huntington’s disease (HD), and amyotrophic lateral sclerosis (ALS) [5,6,7]. Notably, regions of neurodegeneration frequently overlap with areas of iron accumulation, supporting a link between ferroptosis-associated processes and disease pathology [8,9].

Despite growing evidence implicating ferroptosis in neurodegeneration, its precise role remains complex and, in some cases, controversial. While experimental models suggest a causative role for ferroptosis in neuronal injury, clinical observations indicate that ferroptotic features may also arise as secondary consequences of disease progression. This raises important questions regarding the timing, context, and relative contribution of ferroptosis compared to other cell death pathways. Furthermore, variability in neuronal susceptibility across brain regions highlights the need for a more nuanced understanding of ferroptosis in disease-specific contexts.

In this review, we provide a comprehensive overview of ferroptosis in neurodegenerative diseases, with an emphasis on its biological relevance, disease-specific manifestations, and therapeutic potential. We also discuss current advances in detection strategies, therapeutic interventions, and future research directions, highlighting the challenges and opportunities in translating ferroptosis into clinical applications.

## 2. Ferroptosis Mechanistic Drivers

Mechanistically, ferroptosis reflects a failure of lipid peroxide control rather than a single linear pathway. Instead, it emerges from the convergence of iron-dependent lipid peroxidation, dysregulated iron handling, and impaired antioxidant defense systems. The drivers can be grouped into processes that increase lipid peroxide formation and those that weaken their detoxification and repair.

### 2.1. Oxidative Stress and Lipid Peroxidation

Oxidative stress plays a vital role in the induction of ferroptosis. The imbalance between ROS generation and antioxidant levels leads to oxidative stress, triggering the activation of various transcription factors [2]. This oxidative stress often results in oxidation of polyunsaturated fatty acids (PUFA) in membrane phospholipids, which can alter membrane structure and increase membrane permeability [10]. Consequently, the plasma membrane may lose integrity due to the accumulation of lipid hydroperoxides [2]. These peroxides further promote the production of toxic aldehydes, which could inactivate cellular proteins and promote ferroptosis [2]. Oxidative stress drives ferroptosis by amplifying lipid peroxidation and overwhelming cellular antioxidant capacity.

Moreover, ferroptosis can also be triggered by increased intracellular iron availability, which expands the labile Fe^2+^ pool and accelerates ROS-driven lipid peroxidation [11,12]. The nuclear receptor coactivator 4 (NCOA4) mediates ferritinophagy, the autophagic degradation of ferritin, which is the primary cellular iron storage complex [13]. This process releases free iron, potentially increasing intracellular Fe^2+^ concentrations and initiating ferroptosis. Under conditions of oxidative stress, the Fenton reaction generates reactive intermediates that damage membrane lipids and facilitate PUFA peroxidation [12,14]. The Fenton reaction occurs when Fe^2+^ ions react with hydrogen peroxide, producing hydroxyl radicals, which are highly reactive ROS [15]. This production of membrane lipid peroxidation from ROS initiates iron toxicity [12]. Phospholipids containing PUFAs determine the levels of lipid peroxidation and cause subsequent cell death [12].

### 2.2. Antioxidant Defense System

**Figure 1:**
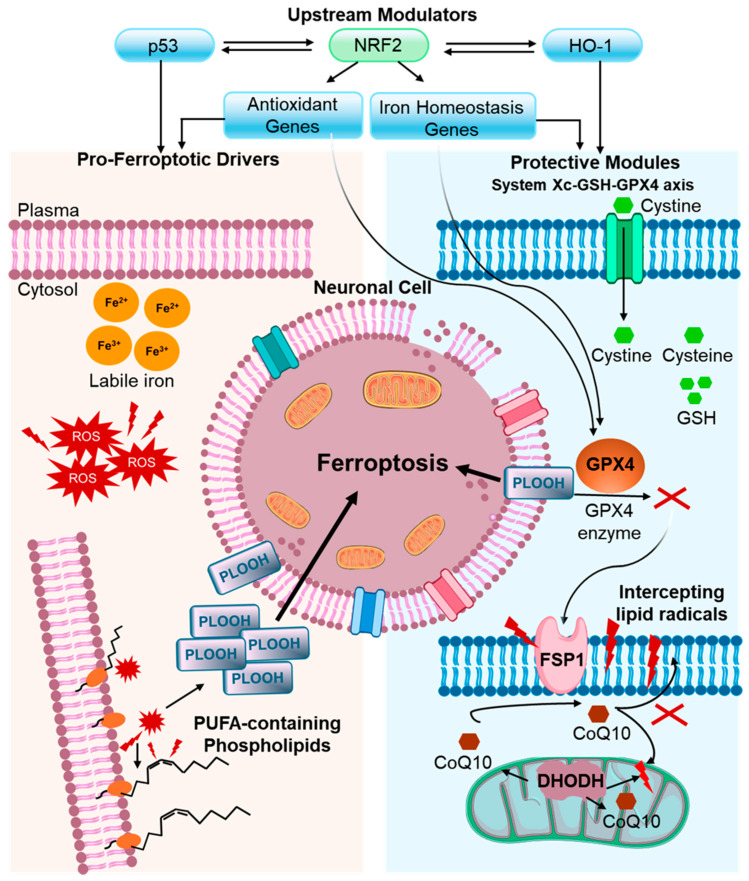
Core ferroptosis circuitry in the central nervous system. Ferroptosis arises from the convergence of labile iron (Fe^2+^), oxidative stress, and PUFA-containing membrane phospholipids, culminating in phospholipid hydroperoxide (PLOOH) accumulation and lethal membrane damage. Protective modules include the System Xc-GSH-GPX4 axis and GPX4-independent CoQ10 defenses (FSP1-CoQ10 at the plasma membrane and DHODH-CoQ10 in mitochondria). NRF2 integrates antioxidant and iron-homeostasis programs, whereas p53 and HO-1 modulate ferroptosis.

Another mechanism involved in ferroptosis induction is system Xc^-^, which is a transport protein that facilitates the exchange of glutamate out of the cell for cysteine import. Cystine is reduced to cysteine, which then forms GSH by glutathione synthase (GSS) to support GPX4 activity [16]. Reduced cystine uptake or impaired GSH synthesis reduces GPX4 activity, leads to increased lipid ROS levels, and thereby promotes ferroptosis [17,18]. A schematic overview of the core ferroptosis circuitry in the CNS, integrating iron imbalance, lipid peroxidation, and the major protective systems (System Xc-GSH-GPX4 and CoQ10-linked defenses), is shown in Figure 1.

### 2.3. Iron Dysregulation and Labile Iron Pool

Iron regulation in the brain is essential as it supports cellular metabolism, mitochondrial respiration, neurotransmitter synthesis, and myelination of neurons [19]. However, excessive or improperly compartmentalized iron can be toxic [12,20]. Iron is transported into the brain via clathrin-mediated endocytosis of Transferrin receptor 1 (TfR1) at the blood–brain barrier (BBB) and on neural cells [21]. Within endosomes, iron is released from transferrin and transported into the cytosol as Fe^2+^ through the divalent metal transporter 1 (DMT1) [9,12]. TfR and transferrin (Tf) facilitate the transfer of Fe^3+^ into brain microvascular endothelial cells from the blood-facing (luminal) side through endocytosis, and the iron is then moved in a controlled manner to the brain-facing (abluminal) side [9]. Astrocytes are well-positioned to take iron from the circulation to redistribute to other cells, as they express machinery required for both iron influx and efflux during cell-to-cell iron transport [22,23]. Iron in the brain circulates in two forms: transferrin-bound (Tf-bound) and non-transferrin-bound [22]. Oligodendrocytes and other cell types can import iron through non-vesicular pathways, often involving DMT1. In contrast, microglia and neurons may take up transferrin-bound iron via transferrin receptors and mediate iron efflux through ferroportin [9]. An imbalance in iron homeostasis increases ferroptosis susceptibility by sustaining a redox-active iron pool [22]. Accordingly, iron uptake, efflux, storage, and turnover are critical processes that shape ferroptosis vulnerability [22]. Moreover, iron is primarily stored in ferritin as Fe^3+^ to mitigate toxicity, especially under conditions of excessive iron [24]. During iron deficiency, ferritin can be degraded by lysosomes to release stored iron and maintain cellular iron availability [12].

## 3. Ferroptosis Vulnerability Factors

Aging worsens ferroptosis, and oxidative stress increases with age; brain ferritin levels rise, often leading to cognitive problems and iron overload. The increase in total iron concentrations with aging is due to various factors, including increased permeability of the blood–brain barrier (BBB), inflammation, and changes in iron homeostasis [9]. A decline in antioxidant capacity, including reduced GSH levels, further increases vulnerability to ferroptotic damage [3,25]. The brain is particularly vulnerable to oxidative damage and ferroptosis due to its high lipid content and oxygen consumption. Age-related iron accumulation may amplify these processes and contribute to disease progression [25,26]. Aging also affects neuromelanin in neurons, promoting the formation and accumulation of neuromelanin-iron complexes across different brain regions [26]. Neuromelanin is present in specific catecholamine neurons and plays a vital role in dopamine production [26]. Furthermore, dopamine’s role in modulating ferroptosis influences neurodegeneration [27]. For example, dopamine has been shown to reduce erastin-induced ferrous iron accumulation, glutathione depletion, and malondialdehyde production. It also stabilizes GPX4, a key enzyme that protects neurons from oxidative stress [27,28].

Ferroptosis has a profound impact on the blood–brain barrier (BBB), primarily through lipid peroxidation, which may disrupt the endothelial membranes [7]. Lipid oxidation processes contribute to BBB dysfunction and increased permeability. CYP enzymes play a regulatory role in astrocytes and function as a metabolic barrier that influences drug influx, vascular tone, and inflammatory signaling [7]. COXs are widely expressed in the central nervous system (CNS) and contribute to BBB disruption through inflammatory pathways and matrix metalloproteinases (MMPs). For instance, lipopolysaccharides (LPS) induce BBB destruction via a COX-dependent pathway, and tumor necrosis factor-alpha (TNF-α) has been shown to enhance BBB permeability by upregulating COX activity and elevating MMP levels. Activated MMPs then degrade tight-junction proteins, which further disrupts BBB integrity [7].

p53 is a critical regulator of cellular stress responses and has been shown to influence BBB permeability [29]. It is located at the center of a signaling network that controls cellular proliferation and death and is mainly activated by DNA damage, nutritional deficiencies, hypoxia, or oxidative stress [30,31]. In a recent study using brain microvascular endothelial cells, p53 was found to support BBB integrity by reducing lipid peroxidation, preserving tight junctions, and limiting oxidative damage [32]. Conversely, increased lipid peroxidation can compromise BBB integrity through accumulation of reactive aldehydes such as malondialdehyde (MDA) and 4-hydroxynonenal (4-HNE) that accumulate and further damage the BBB [32]. p53 can regulate ferroptosis in both pro- and anti-ferroptotic directions, depending on the cell type and stress conditions context, through distinct signaling pathways. On one hand, p53 promotes ferroptosis by repressing SLC7A11 transcription, thereby weakening antioxidant defenses. On the other hand, p53 can inhibit ferroptosis in certain cellular contexts by promoting nuclear localization of dipeptidyl peptidase-4 (DPP4), which reduces lipid ROS levels and ferroptotic sensitivity [33].

## 4. Ferroptosis in Neurodegenerative Diseases

**Figure 2:**
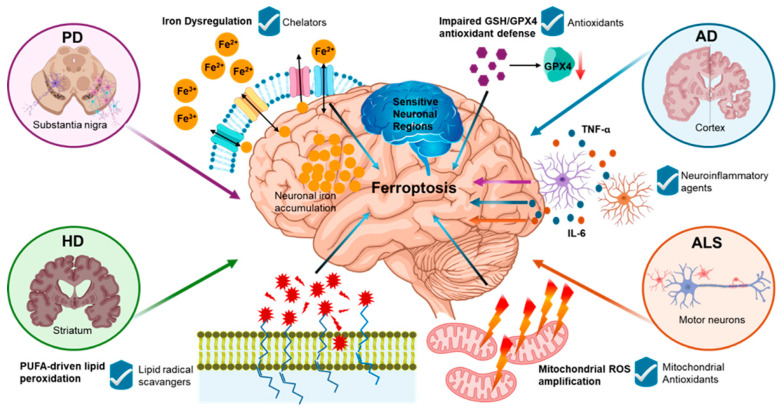
Convergent ferroptosis-associated axes across neurodegenerative diseases. PD, AD, HD, and ALS share overlapping determinants of ferroptotic susceptibility, including iron dyshomeostasis, impairment of GSH/GPX4-linked antioxidant defense, PUFA-driven lipid remodeling and peroxidation, mitochondrial ROS amplification, and permissive neuroinflammatory milieu. Disease context shapes the dominant nodes and vulnerable regions, but the shared axes highlight common therapeutic leverage points that can be targeted to reduce ferroptosis-associated injury.

Iron is essential for mitochondrial respiration, myelin synthesis, and neurotransmitter metabolism, whereas excess redox-active Fe^2+^ can increase oxidative stress and lipid peroxidation, culminating in ferroptosis in neurodegeneration [12,20,34]. Ferroptosis is increasingly implicated in various neurodegenerative diseases, including Parkinson’s disease (PD), Alzheimer’s disease (AD), Huntington’s disease (HD), and Amyotrophic Lateral Sclerosis (ALS) [3]. Although these diseases share ferroptosis-associated features, the relative contribution of iron metabolism, lipid peroxidation, and antioxidant failure varies across disease contexts [35]. In parallel, ROS also plays a significant role in BBB dysfunction by activating lipid-targeting enzymes and signaling cascades, as well as altering tight-junction proteins. Disruption of zonula occludens (ZO) increases BBB permeability, facilitating entry of harmful factors and amplifying neuroinflammation [36]. Experimental models of neurodegeneration have demonstrated that ferroptosis inhibitors and iron chelators can improve outcomes, emphasizing ferroptosis as a potential therapeutic target in disease progression [4,5]. Notably, regions most vulnerable to neuronal loss and atrophy often overlap with sites of iron accumulation, supporting an anatomical link between iron burden and neurodegeneration [9,37]. Excessive iron buildup is also correlated with an accelerated decline in cognitive performance [4]. Figure 2 summarizes shared ferroptosis-related pathways and key therapeutic targets across PD, AD, HD, and ALS.

### 4.1. Parkinson’s Disease

PD is the second most common neurodegenerative disorder, primarily affecting middle-aged and elderly individuals. It is characterized by a gradual decline in motor and non-motor functions due to a significant reduction in dopamine production; PD progresses slowly over time. A key risk factor for PD is iron accumulation in vulnerable brain regions, which contributes to abnormal deposition. Reduced antioxidant capacity further increases neuronal vulnerability. High metabolic demand further enhances susceptibility to oxidative damage [38,39]. A biomarker for PD progression is progressive iron accumulation in the substantia nigra pars compacta (SNpc). MRI scans show that elevated iron signals correlate with both cognitive and motor impairments. Iron accumulates in brain regions, including the globus pallidus, caudate nucleus, premotor cortex, prefrontal lobe, insula, cerebellum, and pons [40]. Also, iron metabolism dysfunction has been observed in the cerebrospinal fluid of PD patients with apathy and REM sleep behavior disorder [41,42]. However, it remains unclear whether iron accumulation acts as a primary driver of neurodegeneration or represents a secondary consequence of neuronal loss. Experimental evidence supports a causative role for iron-mediated damage, whereas clinical observations suggest that iron dysregulation may also arise during disease progression.

Ferroptosis in PD has been tested in various models, both in vitro and in vivo, including differentiated human dopaminergic LUHMES neurons exposed to ferroptosis inducers such as erastin, which display characteristic ferroptosis features [43]. α-synuclein, a major component of Lewy Bodies, plays a central role in PD pathophysiology by linking iron dysregulation and oxidative stress [27,44]. It has been reported to exhibit ferroreductase-like activity, converting Fe^3+^ to Fe^2+^ and binding Fe^2+^, thereby promoting its own misfolding and aggregation into toxic protein assemblies that exacerbate disease progression [45].

Compared to other neurodegenerative diseases, the selective vulnerability of SNpc dopaminergic neurons in PD may reflect a unique convergence of iron accumulation, dopamine metabolism, and oxidative stress.

### 4.2. Alzheimer’s Disease

In AD, dysregulated iron metabolism is a critical factor linking ferroptosis to disease progression [46]. Iron deposition in the brain is accompanied by increased ROS production, mitochondrial dysfunction, and neurodegeneration. High iron levels have been linked with AD severity and disease progression. Accumulated iron can interact with amyloid-β (Aβ) and tau, forming heme-associated complexes that promote oxidative stress and may favor ferroptosis [47]. Tau aggregation into neurofibrillary tangles is also associated with synaptic loss, neuroinflammation, and neuronal death [22]. Heme oxygenase-1 (1HO-1), an oxidative stress-responsive enzyme, has been implicated in iron handling and redox dysregulation in AD models [48]. In mice, HO-1 overexpression promotes iron loading and tau aggregation, thereby inducing AD-like pathological features [22,49]. AD pathology is further characterized by increased lipid oxidation and reduced cortical antioxidant capacity, which together contribute to disease, which together contribute to disease progression [50].

Compared to PD, ferroptosis in AD appears more closely linked to interactions between amyloid-β, tau pathology, and regional lipid metabolic alterations rather than selective neuronal vulnerability alone.

### 4.3. Huntington’s Disease

Huntington’s disease (HD) is an inherited neurodegenerative disease caused by an expanded cytosine-adenine-guanine (CAG) repeat in the huntingtin (HTT) gene, and emerging evidence suggests that ferroptosis contributes to disease progression [51]. In animal models of HD, excessive iron accumulation and oxidative stress have been directly linked to the initiation of ferroptosis [52]. Although no direct interaction is observed between iron and n-terminal HTT fragments, HTT may influence iron by disrupting iron-homeostasis pathways [53]. Consistent with this, mutant huntington (mHTT) is associated with brain iron accumulation in HD, suggesting that mHTT-related metabolic and excitotoxic stress may be vital regulators of Fe status [54,55]. Reduced GSH levels have also been observed in HD, which may further contribute to the neuronal sensitivity to ferroptosis [56]. Increased iron levels were detected in early disease stages in the basal ganglia, occipital cortex, globus pallidus, and putamen by imaging techniques such as MRI and susceptibility mapping [57]. Additionally, arachidonate 5-lipoxygenase (ALOX5), an inducer of ferroptosis, has been implicated in HD models [58]. ALOX5 contributes to ACSL4-dependent ferroptosis and may be promoted by the expression of the N-terminal mHTT polyglutamine fragment (HTTQ94). Notably, loss of ALOX5 expression can prevent HTTQ94-mediated ROS stress and ferroptosis, which may serve as a potential new target for HD [55,59,60].

In contrast to PD and AD, ferroptosis in HD appears to be more strongly associated with mutant huntingtin-driven metabolic stress and lipid oxidation pathways.

### 4.4. Amyotrophic Lateral Sclerosis

Amyotrophic Lateral Sclerosis (ALS) is a neurodegenerative disease that primarily affects motor cells in the CNS, leading to progressive muscle weakness and paralysis [61]. A consistent feature observed in both animal models and patients is iron accumulation in affected regions, particularly the spinal cord and CNS [62,63]. The iron buildup is linked to an imbalance in iron regulation, with increased expression of key iron-handling proteins, including DMT1, TfR1, ferroportin (FPN), and ceruloplasmin (CP), especially in the spinal cord [64]. Oxidative stress plays a significant role in ALS progression, as evidenced by increased lipid peroxidation and protein oxidation markers, notably MDA, 4-HNE, and protein carbonyls in both experimental models and patients [65]. Moreover, neuronal loss of GPX4 leads to motor neuron degeneration and paralysis in animal models; however, its upregulation has been shown to slow ALS progression and improve motor function [66].

Compared to other neurodegenerative diseases, ALS is characterized by a strong link between GPX4 depletion, excitotoxicity, and motor neuron vulnerability. Table 1 provides a comparative summary of key ferroptosis-related features across various neurodegenerative diseases.

### 4.5. Other Diseases

Additionally, other neurodegenerative diseases that are triggered by ferroptosis include epilepsy, brain ischemia, and stroke [83]. In epilepsy, oxidative stress is a prominent pathogenic feature, and the disorder is defined by a persistent predisposition to seizures with neurobiological, cognitive, psychological, and social consequences [84]. Recent evidence shows that oxidative stress and iron dysregulation can act synergistically to exacerbate epileptic cell dysfunction. Thus, high concentrations of unbound Fe^2+^ can catalyze Fenton reactions, converting hydrogen peroxide into highly reactive ROS and thereby promoting neuronal injury [85].

Stroke can be broadly classified as ischemic and hemorrhagic [86]. Ischemic stroke is caused by interruptions of the cerebral blood supply, which accounts for approximately 80% of stroke cases [87]. Hemorrhagic stroke is caused by cerebral vascular rupture and is divided into subarachnoid hemorrhage (SAH), intracerebral hemorrhage (ICH), and intraventricular hemorrhage (IVH) [88]. Both in vitro and in vivo studies support a role of ferroptosis in early brain damage after SAH [89]. After hemorrhage or ischemia, iron released from blood products, together with disrupted iron, can increase brain iron burden. Additionally, BBB disruption can allow iron-rich proteins and ferritin to enter the brain parenchyma, increasing ROS production through Fenton reactions and promoting ferroptotic damage to proteins, membranes, and nucleic acids [90,91].

## 5. In Vivo Detection of Ferroptosis

Ferroptosis in the brain can be visualized using imaging techniques, such as positron emission tomography (PET), magnetic resonance imaging (MRI), and fluorescence-based approaches in both mouse models and humans [92,93]. Because ferroptosis is a molecular process, these modalities primarily detect indirect signatures such as iron burden, redox imbalance, and oxidative damage. Such imaging techniques are most informative when combined with biochemical validation [94].

PET is non-invasive and enables repeated, quantitative molecular imaging of tracer uptake over time [95]. It can probe ferroptosis-related processes by reporting on redox-active iron pools and oxidative stress pathways. For example, the radiotracer 18F-TRX is used to monitor the labile iron pool (LIP), which correlates with the iron-mediated cell death pathways [96]. Another tracer, 18F-labeled dihydromethidine (18F-FDHM), can cross the BBB and react with intracellular ROS, enabling experimental mapping of ROS-rich regions [97].

MRI is widely used in clinical practice and research to monitor the progression of neurodegenerative diseases [93]. MRI technologies use proton transverse relaxation rates to quantify brain iron accumulation, providing indirect but clinically relevant evidence of iron burden in Alzheimer’s and Parkinson’s patients [93]. To improve sensitivity to iron deposition, approaches such as quantitative susceptibility mapping (QSM) and relaxation time mapping have been used to detect iron associated with ferritin and hemosiderin [9]. However, distinguishing Fe^2+^ from Fe^3+^ in routine clinical settings remains challenging, and iron-related signals can be influenced by factors such as myelin changes or calcification [9]. Moreover, MRI-based measurements primarily reflect total iron content and do not directly distinguish ferroptotic activity.

Thirdly, fluorescence imaging is a non-invasive optical imaging method to detect intracellular metabolites, parameters, and biomolecules related to oxidative homeostasis in ferroptosis [98]. Fluorescence probes used in preclinical studies, including in vivo and ex vivo experiments, report intracellular redox alterations and oxidative stress signatures, complementing PET and MRI approaches [99]. One widely used design is the N-oxide reduction strategy, which produces Fe^2+^-fluorescence “turn-on” response [100]. Another probe, BODIPY (4,4-difluoroboradiazaindacene), can specifically detect GSH and image changes in cellular antioxidant status in cells and tissues [101].

A key limitation in the in vivo detection of ferroptosis is the lack of highly specific imaging biomarkers. Most current approaches rely on indirect measures such as iron accumulation or oxidative stress, which overlap with other pathological processes. Therefore, accurate identification of ferroptosis typically requires integration of multiple imaging and biochemical readouts.

## 6. Therapeutic Strategies for Neurodegenerative Diseases

Dopamine has a significant impact on PD as it results from low dopamine levels in the brain. Some of the common dopamine-based therapies include levodopa and dopamine receptor agonists [102]. Levodopa is a dopamine precursor that restores dopamine levels and improves motor symptoms, as supported by established evidence-based treatment guidelines [102]. Moreover, in preclinical models, non-oxidative dopamine has been shown to inhibit erastin-induced ferroptosis, suggesting a protective effect against ferroptotic cell death [103]. Iron chelation therapy is a potential treatment strategy for PD to treat iron overload and protect against neuronal injury. It also prevents dopaminergic neuronal loss in the SNpc and treats motor deficits [104]. A meta-analysis of clinical markers confirms that iron metabolism markers are significantly altered in PD patients, supporting the rationale for iron-targeted interventions [42].

Disruption of antioxidant balance is also associated with dopaminergic neuronal vulnerability in PD [105]. Clinical trials have shown that glutathione administration can restore glutathione levels for mild therapeutic benefits [106]. Alterations in GPX4 activity have been associated with neurodegenerative progression. Loss of GPX4 in dopaminergic neurons has been associated with increased anxiety and diminished spontaneous locomotor activity [107]. A pharmacological inhibitor of ferroptosis uses ferrostatin 1 (Fer-1) derivatives and iron chelators, which have shown improvements in motor control and delays in neurodegeneration. Fer-1 can inhibit 1-methyl-4-phenylpyridinium (MPP+), which kills dopaminergic neurons [108].

In AD, therapeutic strategies primarily focus on modulating oxidative stress and protein aggregation rather than selective neuronal loss. Neuroprotection in AD is often accompanied by strategies that stabilize antioxidant defenses, including production and metabolism of GSH and glutamine, respectively [109]. In addition, aromatic amine antioxidants like Fer-1 and liproxstatin-1 (Lip-1) are potential inhibitors of ferroptosis with demonstrated protective effects in experimental models [110]. α-tocopherol is a form of vitamin E that exerts its antioxidant capacity by interrupting the chain of lipid oxidation [111]. The levels of α-tocopherol in the brain are regulated by the tocopherol transfer protein (TTP), while a deficiency in this protein or vitamin E can exacerbate oxidative stress [111]. Clinical studies have shown that vitamin E supplements can slow cognitive decline and oxidative stress in AD [111]. Nitroxides such as iron (II) citrate can cross the BBB, suggesting therapeutic potential for oxidative stress-induced diseases [22]. Additionally, selenium-based compounds and nanomaterials have been explored for their antioxidant and GPX4-supporting properties. While selenium nanospheres exhibit anti-ferroptotic effects in vitro and in animal models, translational applicability remains uncertain, particularly regarding blood–brain barrier penetration and long-term safety [112]. The small selenium sphere binds to the BBB receptor and targets the αβ42 fibrils in the brain [112]. It transforms αβ42 fibrils into αβ42 oligomers and inhibits tau protein phosphorylation. This treatment reduces neuronal toxicity and ferroptosis and can also be used for other neuroinflammatory diseases [113].

In HD mouse models, iron-selective chelation by using deferoxamine (DFO) or deferiprone (DFP) alleviates disease symptoms [114,115]. Administration of 3-nitropropionic acid (3-NP) can reduce GSH levels, while cysteamine supplementation reverses 3-NP-induced striatal neuronal death by upregulating GSH [115,116]. Fer-1 treatment shows a significant inhibition in lipid peroxidation and iron-induced cell death in HD [114]. In contrast, ALS treatments include ferroptosis inhibitors as potential therapeutic agents. An iron chelator such as DFP is known to improve lifespan in mouse models and has demonstrated potential efficacy in human patients [63,115]. Other iron chelators, such as DFO and SIH, reduce pathological iron accumulation, improve motor neuronal survival, and restore motor function [63]. In ALS, the ferroptosis inhibitor Cull (atsm) prevents lipid peroxidation, and the free radical scavenger, edaravone is found to protect motor neurons and inhibit ferroptosis [117]. Currently, there is no therapeutic intervention available to cure or prevent HD and ALS.

## 7. Ferroptosis-Targeted Neuroprotection

### 7.1. NRF2

The nuclear factor erythroid 2-related factor 2 (NRF2) is a master regulator of cellular antioxidant defense and an important determinant of ferroptosis susceptibility. NRF2 activity is regulated through the KEAP1-NRF2 axis, which controls its stabilization and transcriptional activity under stress conditions [118,119,120,121,122].

A key mechanism underlying NRF2-mediated ferroptosis regulation involves the NRF2-SLC7A11-GPX4 axis. NRF2 upregulates SLC7A11, a component of the system Xc-antiporter, which enhances cystine import and supports GSH synthesis. Increased GSH availability enables GPX4 to detoxify lipid peroxides, thereby limiting ferroptotic damage.

NRF2-mediated neuroprotection is closely linked to the regulation of antioxidants and metabolic pathways that influence ferroptosis susceptibility. Consistent with this, activation of NRF2 signaling has been reported to protect neurons from ferroptosis-like injury in multiple neurodegeneration-relevant models, including traumatic brain injury, intracerebral hemorrhage, and neurotoxin exposure [118,119,120,121,122]. For example, lycopene and silymarin have been shown to mitigate neuronal damage in part by enhancing NRF2-associated pathways [119,121].

### 7.2. Iron Chelators

Iron chelators play a critical role in preventing ferroptosis by removing excess iron from the body. Common iron chelators include DFO, DFP, and DFX, which help regulate iron levels and are safer alternatives that cross the BBB [123]. DFP is a safer alternative to DFO, as it can easily cross the BBB. Conversely, DFX offers protection against kidney and neuronal damage. Some newer iron chelators, CN128 and ciclopirox olamine, are still being studied for broader applications [124].

### 7.3. TFR-1

Another component in the regulation of ferroptosis is TFR-1. Blocking TFR-1 by RNA interference prevents ferroptosis, whereas increasing TFR1 expression stimulates ferroptosis through the NF2-YAP signaling pathways [125]. TFR-1 is also used as a marker for cells that undergo ferroptosis and regulates iron efflux via hepcidin agonists. Additionally, ferroptosis is also targeted by reductive-oxidative pathways through the enzyme HO-1. The enzyme HO-1, encoded by the gene HMOX1, catalyzes heme to produce carbon monoxide and free iron and plays a cytoprotective role against ferroptosis [126]. High HMOX1 can be toxic; it can cause excessive heme breakdown and promote an iron pool. The regulation of ferroptosis by HO-1 is influenced by both HO-1-catalyzed heme metabolites and Nrf2 proteins [127]. In reductive-oxidative responses, gene transcription is activated by NRF2, where the KEAP1-NRF2 axis could be a viable strategy for modulating ferroptosis.

### 7.4. MUFA

Lipid metabolism plays a vital role in ferroptosis. Acetyl-CoA synthetase long-chain family member 4 (ACSL4) promotes phospholipid-PUFA synthesis. Exogenous monounsaturated fatty acids also protect against ferroptosis by reducing lipid ROS accumulation in the plasma membrane and removing PUFAs from their cellular sites [128].

### 7.5. Selenium

Another micronutrient is selenium, which can modulate ferroptosis. It is essential for producing selenocysteine, which acts as the active site for GPX4 [129]. Selenium-containing peptides tat SelPep, can cross the BBB and help protect against ferroptosis-mediated tissue damage.

### 7.6. Nanoparticles

Small-molecule inducers play a significant role in modulating ferroptosis. Ferroptosis was initially defined through small molecules known as RAS-selective lethal compounds (RSLs) [130]. Nanoparticle-based systems can influence ferroptosis-related pathways through targeted delivery of bioactive compounds. For instance, arginine-rich manganese silicate nanoparticles are known to induce ferroptosis in experimental models [131]. Erastin mediates ferroptosis without morphological changes or biochemical processes by inhibiting system Xc^-^ and RSL3 [132]. Ferritin-bound erastin and rapamycin decrease GPX4 activity while enhancing lipid peroxidation. Most nanoparticle approaches to control ferroptosis were first designed for cancer research. For example, SRF@FeIIITA nanoparticles were developed to trigger ferroptosis in tumor models [133]. SRF@FeIIITA, is formed by Fe^3+^ and a network-like tannic acid corona that contains the kinase inhibitor sorafenib (SRF) [133]. Moreover, SRF@FeIIITA releases SRF and reduces GSH levels. They are mentioned here to show that ferroptosis can be controlled using specific treatments; however, these nanoparticles have not been validated for use in neurodegenerative diseases, and significant challenges remain before they can be translated into clinical applications for brain disorders. Other inducers, including buthionine sulfoximine and cisplatin, may induce synthetic lethality like that caused by GSH depletion [134]. Targeting these molecules by ferroptosis inhibitors such as vitamin E, Trolox, DFO, DFX, Zileuton, Ferrostatin-1, and liproxstatin-1 suppresses oxidative stress and prevents ferroptosis cell death [106]. Table 2 summarizes inhibitors and inducers of ferroptosis in neurodegenerative diseases.

## 8. Nutritional Neuroprotection Against Ferroptosis

Diet plays a vital role in preventing ferroptosis by taking multivitamins and consuming antioxidant-rich foods [135].

### 8.1. Multivitamins

Multivitamins have substantial benefits in maintaining physiological functions and lowering toxic effects. Some vitamins have anti-ferroptosis properties, making them an important agent for neuroprotection. Vitamins A, B, C, D, E, and K have been shown to inhibit ferroptosis [135]. Vitamin A is an essential vitamin that provides retinol, retinoic acids, and carotenoids from animal and plant sources. Retinol can resist ferroptosis by directly capturing anti-peroxides. In contrast, carotenoids also prevent ferroptosis by promoting Nrf2 and can mediate signal transduction via retinoic acid receptors [136]. B vitamins are known for their roles in brain function, energy production, NA synthesis, and repair [137]. Vitamin B6 is used to compensate for impaired GSH levels and to restore GPX4 expression, thereby inhibiting ferroptosis. It can also enhance Nrf2 expression and production of antioxidant enzymes [138]. Vitamin C plays its role in oxygenated enzymes, such as the oxidation of Fe^2+^ to Fe^3+^ [138]. It has antioxidant properties that prevent ferroptosis; however, higher concentrations of vitamin C can induce ferroptosis by decreasing GPX4 levels [138]. Additionally, vitamin D is vital in binding to its target organ to complete metabolism and reabsorption. It can inhibit ferroptosis by upregulating the production of anti-ferroptosis proteins and reducing iron accumulation [139]. Vitamin E has lipophilic antioxidants that may protect against excessive degradation. Vitamin E has been shown to protect neurons from oxidative stress-induced damage, positively influencing the prevention and progression of neurodegenerative diseases [79]. Lastly, vitamin K has emerged as a potent endogenous suppressor of ferroptosis. Beyond its classical role in blood coagulation and biomineralization [140], the FSP1-dependent noncanonical vitamin K cycle protects cells against lipid peroxidation and ferroptotic cell death [141]. In the context of neurodegeneration, vitamin K metabolites have been shown to inhibit ferroptosis in neuronal models, suggesting a protective role against oxidative damage [142].

### 8.2. Jucara Fruit Extract

Jucara fruit extracts exert neuroprotection against glutamate-induced oxidative stress in HT22 cells [143]. These cells are immortalized and are used to study the neuroprotective effects against glutamate-induced oxidative stress. Jucara extract contains high concentrations of antioxidants. The phenolic compounds were found in fractions of crude extracts as well as in hexane, dichloromethane, ethyl acetate, and butanol. These fractions tested the viability of HT22 cells during co-treatment. The results showed that dichloromethane and hexane fractions from fruit extraction protect HT22 cells through their phenolic compounds. The results are still being studied to determine the neuroprotective dosing regimen of Jucara fruits [143,144].

### 8.3. Flavonoids

Flavonol fisetin is effective in preventing ferroptosis in preclinical models [145]. There is evidence that 2 out of the 30 flavonoids can maintain GSH levels in oxidative stress. Fisetin is abundant in fruits and vegetables; high amounts are found in strawberries (160 µg/g), and low amounts in apples, persimmons, kiwis, peaches, grapes, tomatoes, onions, and cucumbers. The bioavailability of flavonoid compounds is yet to be studied [146]. Experiments on mice have been conducted to determine the effects of Fisetin on neurodegenerative diseases. In AD, fisetin consistently prevents cognitive decline [147]. It also maintains synaptic proteins and decreases markers of inflammation and oxidative stress. In PD, fisetin improves motor function and reduces rotenone-mediated decrease in dopamine levels and immune reactivity [148,149]. Moreover, fisetin also improves mitochondrial function and markers of oxidative stress in the midbrain. In HD, mice on a fisetin-based diet had a slower decline in motor function than those on a regular diet; however, further research on the underlying effects and observations is still needed [150]. Thus, flavonoids are promising neuroprotective compounds that require additional research to confirm these effects [149].

## 9. Clinical Translational Challenges

Despite encouraging preclinical data, clinical application of ferroptosis-targeted therapies in neurodegeneration remains limited. Iron chelation is one of the most clinically explored approaches, yet results have been inconsistent. The FAIR-PARK-II trial (NCT02655315), a randomized, placebo-controlled study, tested the iron chelator deferiprone in early-stage Parkinson’s disease [77]. Over 36 weeks, the study found no significant difference in clinical progression between the deferiprone and placebo groups, although substantia nigra iron-related MRI signals were reduced in the deferiprone arm [77]. Systemic iron chelation also raises safety concerns, including anemia and disruption of essential iron-dependent physiology [151]. Future directions may therefore require targeted delivery strategies or chelators with improved selectivity for pathological brain iron pools.

Beyond iron chelation, relatively few clinical trials in neurodegeneration are explicitly designed to target ferroptosis [152]. There remains a clear need for ferroptosis inhibitors that cross the blood–brain barrier and retain sufficient selectivity in vivo. Progress is also limited by the absence of validated biomarkers to track ferroptosis in humans, making it challenging to confirm target engagement and evaluate therapeutic efficacy in clinical studies [153]. Future research should focus on developing and validating accessible biomarkers (CSF or plasma) reflecting lipid peroxidation and antioxidant capacity that are relevant to ferroptosis and can be interpreted alongside iron and redox measures [153].

## 10. Current Limitations and Controversies

Although interest in ferroptosis as a contributor to neurodegeneration has expanded rapidly, several limitations and unresolved issues must be addressed to strengthen its translational relevance. Current mechanistic understanding is largely derived from cellular and animal models, whereas direct evidence across different disease stages in human patients remains comparatively limited [36,154].

A major challenge is the reliable discrimination of ferroptosis from other regulated cell-death programs in vivo. Neurodegenerative diseases reflect a complex convergence of cell-death mechanisms, including apoptosis, necroptosis, and autophagy, which are not mutually exclusive and may be activated concurrently or sequentially [155]. These pathways share upstream stressors and downstream features, complicating mechanistic attribution. For example, oxidative stress and mitochondrial dysfunction are prominent drivers in both ferroptosis and apoptosis [155]. Moreover, morphological characteristics associated with ferroptosis, such as mitochondrial condensation, reduced mitochondrial size, and increased membrane density, can be subtle and context-dependent, particularly in post-mortem human brain tissue [155]. This overlap limits the ability to assign neuronal loss to ferroptosis conclusively and to quantify its independent contribution to overall disease pathology.

In addition, the extent and mechanistic basis of ferroptosis involvement appear to vary across neurodegenerative disorders. Although iron dyshomeostasis is a recurring feature, the proximal triggers and dominant drivers of ferroptosis may differ substantially among Parkinson’s disease, Alzheimer’s disease, and amyotrophic lateral sclerosis [9]. For instance, α-synuclein-iron interactions have been proposed as an amplifying mechanism in Parkinson’s disease [68]. In contrast, in Alzheimer’s disease, ferroptosis-relevant processes may be shaped more strongly by the interplay among amyloid-β pathology, tau aggregation, and regional iron accumulation [9]. Heterogeneity in experimental models, genetic backgrounds, outcome measures, and disease stages examined likely contributes to conflicting observations [5]. A more standardized and critical assessment framework is therefore needed to define when and where ferroptosis is most relevant within each disorder.

Another key limitation is the scarcity of reliable in vivo evidence for ferroptosis in human patients. Most supporting data originates from experimental systems, while clinical measurements often capture correlated processes rather than ferroptosis itself. Although MRI can detect regional iron accumulation, these readouts are not specific and may also reflect inflammation, aging, or broader disturbances in iron metabolism [9]. Progress will depend on the development and validation of sensitive, clinically feasible biomarkers that more accurately reflect ferroptosis biology and can be measured in accessible matrices, such as cerebrospinal fluid or blood [156]. Although lipid peroxidation products such as 4-HNE and MDA are commonly used as indicators, they lack specificity and should be interpreted in combination with complementary biochemical and imaging readouts [157]. The current lack of validated in vivo biomarkers limits the ability to monitor ferroptosis activity in humans, assess target engagement in clinical trials, and establish robust causal links between ferroptosis and neurodegenerative disease progression [5]. Despite promising preclinical findings, therapeutic strategies targeting ferroptosis-related pathways remain limited. Key challenges include poor blood–brain barrier penetration, disruption of physiological redox balance, and variability across disease stages [152].

## 11. Future Technologies

Although multiple techniques have been proposed to mitigate ferroptosis, there are still research gaps to be bridged across mechanistic insights to safe translational approaches. New technologies and methodologies are being explored to enhance the understanding of ferroptosis in neurodegeneration.

Targeted protein degradation is an emerging therapeutic approach that eliminates “undrugged” targets and other difficult-to-treat proteins [158]. Three major classifications of protein degraders are: proteolysis-targeting chimeras (PROTACs), monomeric targeted protein degraders, and molecular glues (MGs). PROTACs are small heterobifunctional molecules that have a target-binding ligand and an E3 ubiquitin ligase-binding ligand connected by a linker to promote target ubiquitination and protein degradation [158]. On the other hand, monomeric degraders have a lower molecular weight and can easily cross the BBB. MGs induce proximity between the target protein and the ubiquitin ligase, which causes protein degradation. Future studies are needed to determine the efficacy and safety of monomeric targeted protein degraders or MG-based degraders to target ferroptosis-related proteins [158].

Artificial intelligence (AI) has the potential to revolutionize drug discovery by improving predictive accuracy and expediting drug development [159]. Rapid machine-based decision-making using artificial neural networks can serve as a cost-effective platform to identify new drugs [159]. AI can integrate bioinformatics and pharmacological networks to accelerate the discovery of new therapeutics to target ferroptosis.

Melatonin, which is produced in the pineal glands, has anti-aging, anti-inflammatory, and anti-cancer properties [160]. It was recently determined that melatonin can affect anti-ferroptosis pathways, including Nrf2 antioxidants, GPX4, HO-1, and NCOA4 [161]. It can inhibit ferroptosis by activating intracellular defense regulatory pathways. Melatonin can suppress inflammation and ROS generation and can aid in regulating autophagy and apoptosis pathways. Further research is needed to elucidate the exact mechanisms by which melatonin can inhibit ferroptosis and be a potential therapeutic target [162].

RNA-based therapies represent a rapidly expanding field primarily using messenger RNA (mRNA), RNAi, single-stranded antisense oligonucleotides, aptamers, ribozymes, and CRISPR-Cas endonuclease-mediated gene editing [163]. These technologies are used to develop vaccines and are now being explored for the treatment of various diseases, including ferroptosis. RNA-based therapies could offer a new therapeutic approach to prevent ferroptosis; however, the research in this area remains in its early stages [163].

## 12. Conclusions

Ferroptosis is an iron-dependent cell death characterized by excessive lipid peroxide accumulation. With the current understanding of oxidative stress and its impact on neurodegeneration, it is determined that ferroptosis plays a significant role in causing neurodegenerative diseases like PD, AD, HD, and ALS. However, current solutions and implications require more specification, validation, and an efficient delivery route to reach a particular brain region to preserve iron metabolism while limiting toxic lipid peroxidation.

## Figures and Tables

**Table 1:** Comparative Summary of Ferroptosis-Related Features in Neurodegenerative Diseases.

Feature	Parkinson’s Disease	Alzheimer’s Disease	Huntington’s Disease	Amyotrophic Lateral Sclerosis
**Iron Dysregulation Pattern**	Accumulation in substantia nigra, globus pallidus, and caudate nucleus [67,68].	Deposition associated with amyloid-β and tau aggregates [69].	Accumulation in the basal ganglia, occipital cortex, and putamen [70].	Accumulation in the spinal cord and motor cortex; altered expression of iron transporters (DMT1, FPN) [71,72].
**GPX4/GSH Status**	Decreased GSH levels; loss of GPX4 in dopaminergic neurons [8,12].	Reduced cortical GSH and GPX4 levels [6,15,18].	Reduced GSH levels. [25,27].	Neuronal loss of GPX4; upregulation slows disease progression [73].
**Lipid Peroxidation Markers**	Increased lipid peroxidation, though specific markers are not always detailed [7,8,11].	Increased lipid peroxides (e.g., 4-HNE, MDA) and lipid ROS [15,74,75].	Increased oxidative stress markers. [25,70]	Increased MDA, 4-HNE, and protein carbonyls [74,75].
**Key Ferroptosis Drivers**	α-synuclein aggregation, dopamine oxidation, mitochondrial dysfunction [68].	Amyloid-β and tau pathology, heme oxygenase-1 (HO-1) activity [69].	Mutant huntingtin (mHTT) expression, excitotoxicity, and ALOX5 activity [70].	SOD1 mutations (in familial ALS), glutamate excitotoxicity, and mitochondrial dysfunction [72].
**Therapeutic Targets**	Iron chelators (e.g., deferiprone), dopamine agonists, and antioxidants (e.g., Fer-1) [76,77,78].	Iron chelators, antioxidants (e.g., Vitamin E), selenium compounds [79,80].	Iron chelators (e.g., DFO), ALOX5 inhibitors, mitochondrial protectors [81].	Iron chelators (e.g., DFO, SIH), radical scavengers (e.g., edaravone), and ferroptosis inhibitors [82].

**Table 2:** Inhibitors and inducers of ferroptosis in neurodegenerative diseases.

Mechanisms	Purpose	Method Examples	Role of Action	References
Dopamine-based therapies	Inhibitor	Levodopa, a dopamine receptor agonist	In PD, these drugs can cross the BBB, facilitate the removal of excess iron from the brain, and stabilize GPX4.	[102]
TfR 1 regulators	Inhibitor	Hepcidin agonist	In AD, regulating TfR 1 mediates cellular iron uptake and maintains iron homeostasis in neuronal cells.	[125]
Antioxidants	Inhibitor	Fer-1, Lip-1	It targets lipid peroxidation and slows cognitive decline in patients with mild-to-moderate AD. It blocks ROS and effectively fixes AB-induced neuronal death	[110]
ROS free radicals	Inhibitor	Edaravone	In ALS patients, it reduces motor neuron damage and inhibits ferroptosis.	[117]
Iron Chelators	Inhibitor	DFP, DFO, DFE	In PD, it protects against neuronal injury through inhibiting ferroptosis. In AD, DFO inhibits erastin-induced ROS accumulation. In ALS, it is shown to improve motor neuron survival and restore motor function. In HD, IV administration of DFO is shown to relieve symptoms in mouse models.	[114,115,123]
Vitamin E	Inhibitor	A-tocopherol	It targets lipid peroxidation and slows cognitive decline in patients with mild-to-moderate AD. It destroys the chain reaction of automatic oxidation.	[111]
Nitroxides	Inhibitor	NOX2 mediated ROS, Iron (II) citrate	In neurogenerative diseases, nitroxides can cross the BBB and target lipid peroxidation. In AD, nitroxides positively induce neuroplasticity and neuroprotection.	[22]
Selenium	Inhibitor	Selenocysteine, Tat SelPep	It acts as the active site of GPX4 and can cross the BBB to help protect against ferroptosis.	[112]
Zileuton	Inhibitor	5-lipoxygenase (LOX)	It protects cells from lipid peroxidation by down-regulating LOX.	[108]
Erastin	Inducer	System Xc^-^	It mediates ferroptosis via inhibiting system Xc^-^.	[132]
Glutamate	Inducer	cystine	It mediates ferroptosis through cystine uptake inhibition of system Xc^-^.	[16]
Sulfasalazine	Inducer	System Xc^-^	It mediates ferroptosis via inhibiting system Xc^-^.	[16]
Sorafenin	Inducer	cystine	It mediates ferroptosis through cystine uptake inhibition of system Xc^-^.	[14]
RSL3	Inducer	Selenocysteine	It blocks the activity of GSH and GPX4 at the active site selenocysteine.	[8]

## Data Availability

Data sharing is not applicable to this article as no new datasets were generated or analyzed.
